# Educational Intervention and Melanoma Prognosis in Black People

**DOI:** 10.7759/cureus.49527

**Published:** 2023-11-27

**Authors:** Rahila Shaikh, Vaughn E Huckfeldt Jr

**Affiliations:** 1 Dermatology, Internal Medicine, Saba University School of Medicine, The Bottom, BES; 2 Epidemiology and Public Health, Saba University School of Medicine, The Bottom, BES

**Keywords:** white, people, black, cancer, prognosis, sun protective measures, ­skin cancer, educational intervention, diagnosis, melanoma

## Abstract

A systematic search was conducted on the PubMed database using the keywords "education," "melanoma," and "African Americans." The results were filtered to include studies published within the last decade that focused on melanoma diagnosis, risk, or outcomes. These selected studies included African Americans, non-Hispanic Black individuals, or those with darker skin tones and compared them with educational interventions or non-Hispanic White groups. The studies showed an overall positive impact of targeted educational initiatives on using sun-protective behaviors, health education, and self-efficacy related to melanoma. Notably, interventions proven to be effective for non-Hispanic White populations also demonstrated efficacy in other racial groups, including non-Hispanic Blacks. It was observed that non-Hispanic Blacks exhibited an overall reduced level of concern regarding melanoma, partly due to its lower incidence within this demographic. Furthermore, these studies focussed on the disparities in melanoma incidence and survival rates among different racial groups. Non-Hispanic Black individuals were found to have a higher incidence of melanoma and lower survival rates compared to non-Hispanic White individuals. Collectively, the studies evaluating melanoma educational interventions for darker skin tones yield promising results for improving melanoma prognosis in Black communities. They underscore the importance of addressing racial disparities in melanoma awareness, diagnosis, and treatments. These studies also highlighted the barriers to sun protection behaviors, such as cost, limited awareness, and misconceptions, particularly prevalent among ethnic communities and youth. The use of melanoma photographs specifically tailored to darker skin tones in outreach efforts to enhance identification, self-examination, and early detection should be considered to improve melanoma prognosis similar to the non-White population.

## Introduction and background

Cutaneous melanoma is the quickest-growing type of skin cancer that is linked to inadequate sun protection and is responsible for 75% of skin cancer-related deaths [1,2]. Melanoma incidence rates vary significantly by race, with Black individuals experiencing the lowest annual incidence at 2.3 per 100,000, while White individuals exhibit the highest incidence at 18.4 per 100,000 [3]. When it comes to five-year survival rates for melanoma, there is a notable difference, with Black individuals having the lowest rate at 72.2%, and White individuals the highest at 89.6% [3,4]. Additionally, Black individuals often receive diagnoses at more advanced stages of melanoma compared to White individuals [5]. This discrepancy in both survival rates and stage at diagnosis may indicate delayed detection of melanoma among Black individuals, which can have a negative impact on their prognosis [6].

Cutaneous melanoma is a melanocyte neoplasm, originating from cells that naturally reside in the basal layer of the skin's epidermis, where they contribute to skin pigmentation [1]. This type of cancer can emerge when ultraviolet rays, whether from sunlight or artificial sources such as tanning beds, penetrate the skin's protective barrier, resulting in DNA damage and cellular mutations [7]. Prolonged or heightened exposure to ultraviolet rays amplifies the likelihood of DNA damage and mutation development, consequently elevating the risk of cutaneous melanoma. Cutaneous melanoma comprises various histological subtypes, and these subtypes exhibit similar histological characteristics across all racial backgrounds. However, their appearance varies distinctly on different skin tones, posing a challenge for visual differentiation. 

Black individuals often perceive themselves as having a lower risk of melanoma, possibly due to the comparatively lower incidence rates when compared to White individuals. Unfortunately, these misconceptions can result in a reduced frequency of skin self-examinations [8-11]. Furthermore, there is a notable lack of melanoma awareness and risk perception, coupled with a lack of educational initiatives specifically tailored to darker skin tones, both within the general public and among healthcare providers. This shortage of educational interventions for individuals with dark skin tones contributes to elevated morbidity and mortality in Black communities, partly due to delayed melanoma diagnoses [12]. Additionally, there is limited knowledge and awareness among both the public and healthcare professionals regarding the atypical presentations of melanoma in individuals with darker skin tones, which increases the risk of melanoma being diagnosed at more advanced stages [5]. Information sources related to melanoma in Black communities are sparse and fragmented, with physicians often not serving as the primary resource for melanoma education [13]. Regrettably, specific guidelines for counseling and educating Black individuals on ultraviolet protection are lacking [14]. As a result, melanoma tends to manifest more severely and is typically discovered at later ages and in more advanced stages among Black individuals compared to their White counterparts [2,12]. This discrepancy can be attributed, in part, to the limited availability of melanoma education materials tailored to darker skin tones, including a scarcity of photographs representing such individuals in melanoma education materials and videos. Visual aids in educational campaigns have proven effective in promoting skin self-examinations, and photographic resources have been correlated with reductions in melanoma thickness at initial presentation [15].

White individuals generally benefit from a more extensive array of melanoma educational resources, which often feature a broader spectrum of melanoma stages visually represented on their skin tone in various websites and informational pamphlets. This, in turn, contributes to greater melanoma awareness among White individuals within both the general population and the healthcare sector. The positive outcomes of these enhanced educational efforts are evident in the higher melanoma survival rates and earlier diagnosis stages observed among White individuals, despite their increased incidence rates compared to Black individuals. To bridge this gap and improve melanoma awareness, risk perception, and self-examination effectiveness in Black individuals, it is imperative to expand the availability of photographs depicting melanoma stages on darker skin tones. Such inclusivity in educational materials will not only aid in the identification of melanoma but also underscore its relevance for Black communities [13].

This paper delves into recent studies focusing on melanoma diagnosis, risk assessment, and prognoses within Black communities, comparing the impact of melanoma educational interventions. The aim is to elucidate the significance of these interventions, equipping healthcare professionals to more effectively educate the public about melanoma in individuals with darker skin tones. Furthermore, the hope is that these efforts will contribute to the early detection of melanoma among Black individuals, thereby reducing health disparities and enhancing melanoma prognosis and survival rates. With early detection, there is potential for a cure [2].

This article was originally presented as a poster at the 2023 SRGC Annual Conference, held on November 9-10, 2023.

## Review

Methods

A PubMed database structured search was carried out to include MeSH terms and synonyms.

Search string: search strategy (databases to be searched; keywords to search; inclusion/exclusion criteria (i.e., publication dates range; study population(s); criteria for assessing the quality of a study, etc.)

Keywords: (Education [mh] OR Education* OR Health Knowledge, Attitudes, Practice [MH] OR Health Education [MH] OR Health Literacy [MH] OR Health Behavior [mh] OR Health Promotion [MH]) AND (Melanoma [MH] OR Melanoma [tw] OR Skin Neoplasms [MH]) AND (African Americans [MH] OR "Black American" OR Black people*) NOT (review [pt] OR Systematic Review [pt] OR Meta-Analysis [pt]) 

**Figure 1:**
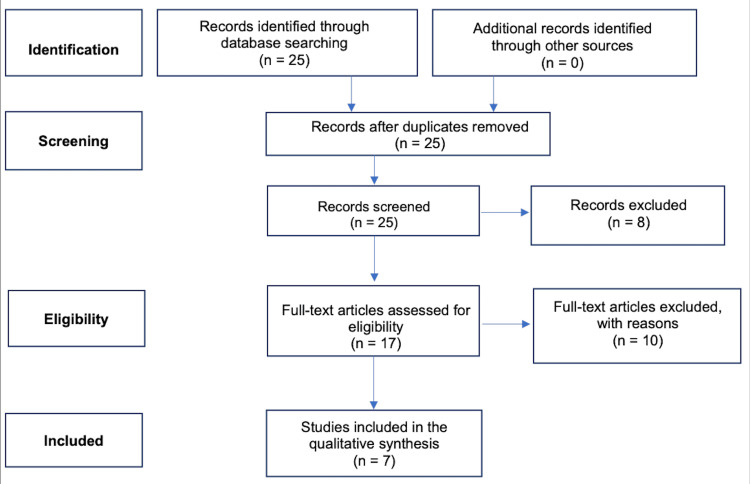
Identification and screening of studies for inclusion in this review The PRISMA Statement for Reviews [13].

Following a comprehensive search and subsequent filtering process for studies published in the past decade (2013-2023), a total of 25 articles were identified, of which one was a randomized controlled trial. Subsequently, the 25 articles underwent a rigorous assessment to determine their adherence to the inclusion criteria. The studies included in this review adhered to specific inclusion criteria, which dictated that they must have been published within the past decade and focused on the assessment of melanoma diagnosis, risk, or outcomes. Additionally, the study population had to encompass individuals of African American, non-Hispanic Black, Black, or Dark skin tone, and they should have incorporated an educational intervention as a key component of the study. Furthermore, in some cases, a non-Hispanic White comparison group was deemed essential. This filtration resulted in the retention of seven studies that met all the specified criteria. A visual representation of the screening and selection process employed in this review is presented in Figure 1.

Results

This review encompassed seven distinct studies that met the specified inclusion criteria, with none being a meta-analysis or systematic review. Notably, only one of these seven studies employed a randomized controlled trial design. The methodology and findings of each of these seven studies are presented below, categorized by their respective study designs. Additionally, a comprehensive summary of the level of evidence, the study population, the therapeutic interventions or exposures investigated, and the study outcomes are available in the evidence table (Table 1).

**Table 1:** Evidence table for the studies Note: Information in this table was sourced from each full-text article.

First Author	Date of Publication	Study Design	Level of Evidence	Study Population	Therapy or Exposure	Outcome/Results
Tsai et al. [15]	April 2018	RCT	1	Self-identified African Americans, at least 18 years old, fluent, and literate in English	The brochure-intervention group received a melanoma brochure, and the video-intervention group received a brochure and watched an online melanoma tutorial.	Equal improvement from pre- to-post education about sun-protective behaviors, knowledge, and self-efficacy about melanoma in both intervention groups.
Chao et al. [16]	June 2017	Educational intervention with surveys and questionnaires	2	70 participants who self-identified as African American, Asian, or Hispanic were recruited following their dermatology visit	Comparison intervention group: an educational intervention using a conventional pamphlet on the 'ABCDEs' of melanoma. Targeted intervention group: modified pamphlet for darker skin of color. Completed a 2-month follow-up questionnaire.	Increase in self-skin examinations after targeted educational intervention towards people of color.
Culp et al. [17]	June 2019	Educational case studies	3	Non-Hispanic Black US population	US Cancer Statistics 2001-2015 Public Use Research Database to examine melanoma incidence and 5-year survival.	Incidence and survival rates in non-Hispanic Black people are lower than in the non-Hispanic white population.
Dawes et al. [18]	November 2016	Comparative cohort study	3	White, Hispanic, Asian American, Native American, Pacific Islander, Black	Surveillance, Epidemiology, and End Results database was used to populate patients given the diagnosis of cutaneous melanoma as their primary cancer from 1992 to 2009.	Lower incidence and survival rates for cutaneous melanoma in non-White population.
Amber et al. [19]	March 2015	Observational study without control	4	Adult beachgoers	10-item Survey (2 different pictures with identical skin conditions in individuals with Fitzpatrick skin types I-IV).	African American population scored melanoma photographs as being of significantly less concern than participants of other racial groups.
Chapman et al. [20]	November 2015	Case reports	4	150 students (from 3 middle schools) in South Los Angeles with predominant Latino and African American youth	Pre-intervention survey and review dermatologic health literacy results, “core” learning concepts about sun safety, full-day education on skin disease and sun protection from an early age, and post-intervention assessment with 3-month follow-up.	Intended compliance to sun protection occurred post-educational intervention.
Wyant et al. [21]	Dec 2022	Cross-sectional	4	859 Florida College marching band members and alumni	Anonymous cross-sectional web-based surveys were distributed via email and private Facebook groups to assess demographic characteristics, sun protection behaviors, as well as history of sunburn and skin cancer	Melanoma diagnoses were only reported by alumni who self-identified as non-Hispanic white; none of the non-Hispanic black, Hispanic, or other alumni reported a melanoma diagnosis.

Of importance, all seven studies focused on Black individuals as their primary study population, aligning with the inclusion criteria for this review. Notably, four out of the seven studies extended their scope to include non-Hispanic White individuals as a study population. In comparison, the remaining three studies specifically centered on educational interventions and their comparisons within the Black population. Among these studies, three were primarily oriented toward assessing the impact of educational interventions. They achieved this by comparing pre-survey questionnaire results with post-intervention follow-up questionnaire findings. Furthermore, two studies employed surveys and questionnaires to gauge individual perceptions. Lastly, two studies harnessed extensive databases, notably the US Cancer Statistics 2001-2015 Public Use Research database and the National Cancer Institute Surveillance, Epidemiology, and End Result database. These databases served as the primary source of data for the research, facilitating comparisons between melanoma incidence and survival rates among Black and White individuals. The investigations considered melanoma cases diagnosed either before the individuals' demise or as their primary cancer diagnosis, respectively.

Clinical Trial 

In the study conducted by Tsai et al. [15], a comprehensive assessment was carried out involving 143 participants. Their research encompassed a pre-survey questionnaire to gauge sun-protective behavior, followed by a randomized controlled trial involving two different melanoma education interventions. These interventions, involving either a brochure or video, were administered immediately following the pre-survey. Subsequently, a post-survey questionnaire was administered, and this process was repeated once more at a one-month follow-up. It is worth noting that all participants in this study self-identified as African Americans and did not seek clinical attention for skin-cancer-related issues.

The study employed a customized tool, though its specific name was not provided in the study, which was tailored based on its performance among the African American population. This tool was used to generate composite scores for both pre-survey and post-survey assessments related to photoprotection. The interpretation of these scores was carried out using a 5-point Likert scale, with a Cronbach alpha reliability coefficient of 0.74. Analysis of the results revealed that the composite scores for sun-protective behaviors showed no statistically significant improvement, with a pre-survey score of 1.85 and a post-survey score of 2.07 (p=0.16) for the control intervention group. However, the target intervention group did exhibit a statistically significant improvement, with scores moving from 1.78 to 2.08 (p=0.001).

The one-month follow-up showed an overall participant retention rate of 53%. The control group exhibited a follow-up rate of 47% (34 participants), whereas the target intervention group had a higher rate of 59% (42 participants). For the control intervention group, a statistically significant increase (p≤0.001) was observed in all categories, except for the sun-protective behaviors category (p=0.16). In contrast, the intervention group demonstrated a statistically significant increase (p<0.05) in all composite scores for the categories assessed. It is noteworthy that a direct comparison of composite scores between the control and intervention groups was not conducted within this study [15].

Non-randomized Trial with Controls

In their study, Chao et al. [16] engaged a cohort of 100 participants who completed a pre-intervention questionnaire and then were exposed to one of two scripted melanoma educational interventions. Subsequently, they participated in a post-intervention questionnaire, immediately following the intervention, and then once again at a two-month interval. All participants in the study self-identified as African American, Asian, or Hispanic and were above 18 years of age.

These participants were alternately assigned to either the comparison or target intervention groups. The comparison intervention group received a conventional pamphlet explaining the "ABCDEs" (Asymmetry, Borders, Color, Diameter, Evolution) of melanoma, derived from the Skin Cancer Foundation brochure. On the other hand, the target intervention group received a modified pamphlet, also sourced from the Skin Cancer Foundation, which incorporated a dedicated section for skin of color and included the title "For All Races and Skin Colors." It introduced the term "melanoma skin cancer" and included an image demonstrating a skin self-examination, where an individual sought the assistance of a friend for a foot examination.

The immediate post-intervention assessment revealed a statistically significant increase in the perceived risk of developing melanoma in the comparison intervention group compared to the target intervention group. However, this statistical significance ceased to be evident between the two groups at the two-month follow-up. Both groups demonstrated a statistically significant enhancement in knowledge concerning skin examination warning signs and the practice of skin self-examination, evident at both the pre-intervention and the two-month post-intervention assessments. Additionally, the target intervention group exhibited a statistically significant increase in the examination of areas such as the mouth, lips, palms, and the soles of the feet when comparing the pre-intervention and the two-month post-intervention assessments.

Observational Studies with Controls

In their study, Culp et al. [17] conducted an analysis using the US Cancer Statistics 2001-2015 Public Use Research Database to investigate melanoma incidence and five-year survival rates among non-Hispanic Black individuals. Between 2011 and 2015, 1,795 non-Hispanic Black individuals were diagnosed with melanoma, and the melanoma incidence among this group was one per 100,000, with the incidence rate increasing with age. Notably, men aged 65 or older exhibited an elevated rate of 5.5 per 100,000, compared to women's rate of 4.1 per 100,000, with statistical significance observed for both sexes. Furthermore, men were less likely than women to receive a diagnosis at a localized stage, which accounted for 53.6% of all melanoma diagnoses within the non-Hispanic population overall. Melanoma was most commonly observed on the lower extremities, particularly the legs and feet, among non-Hispanic Black participants (48.2%). Acral lentiginous melanoma was the most commonly diagnosed subtype among non-Hispanic Black individuals, accounting for 16.7% of cases, following cases classified as having "unspecific histology" (63.8%). Comparatively, a larger proportion of non-Hispanic White individuals (78%) received diagnoses of localized melanoma, in contrast to non-Hispanic Black individuals (55%). Fewer non-Hispanic White participants (9%) were diagnosed with regional-stage melanoma compared to non-Hispanic Black individuals (18%). Notably, a greater percentage of non-Hispanic Black individuals (16%) received diagnoses of distant melanoma metastasis, as opposed to non-Hispanic White individuals (5%). Additionally, more non-Hispanic Black individuals (10%) received diagnoses categorized as "not staged" in comparison to non-Hispanic White individuals (8%). Superficial spreading melanoma was more frequently diagnosed in non-Hispanic White individuals (79%) compared to non-Hispanic Black individuals (29%). Nodular melanoma was more prevalent in non-Hispanic Black individuals (25%) than in White individuals (19%). Intriguingly, non-Hispanic Black individuals exhibited a higher prevalence of acral lentiginous melanoma (46%) compared to non-Hispanic White individuals (2%), despite the incidence of acral lentiginous melanoma being similar between the two groups. In terms of five-year melanoma survival rates, non-Hispanic Black individuals exhibited a lower rate at 66.2%, in contrast to non-Hispanic White individuals, who had a significantly higher rate of 90.1%.

In their study, Dawes et al. [18] harnessed the National Cancer Institute Surveillance, Epidemiology, and End Result database to establish a retrospective cohort spanning from 1992 to 2009, comprising 96,953 patients diagnosed with cutaneous melanoma as their primary cancer. Of this cohort, 91,572 were White patients, and 509 were Black patients. The staging system employed in this study was devised by amalgamating the American Joint Committee on Cancer criteria for stage presentation with the Breslow depth, nodal involvement, and ulceration of the melanoma at the time of diagnosis, resulting in a staging system ranging from 0 to IV. The primary melanoma incidence rate was notably higher among White patients, with a rate of 45.8 per 100,000, compared to Black individuals who exhibited a lower incidence rate of 1.35 per 100,000. Within the White patient group, the majority of patients were diagnosed at stage I (75.9%), and they exhibited the lowest proportion of diagnoses at later stages of melanoma. In contrast, Black patients displayed a lower proportion of diagnoses at stage I (52.6%) and a higher proportion diagnosed at stages II-IV. As the stages progressed, Black patients exhibited an elevated relative risk (RR) compared to White patients, with stage IV showing the highest increase in RR, reaching 2.49. The survival rates among Black patients were notably lower, with a rate of 30% in stage I and 20% in stage III, compared to White patients who exhibited significantly higher rates of 65% and 45%, respectively. Additionally, Black patients displayed higher hazard ratios for mortality across all stages of melanoma when compared to White patients. Overall, White patients demonstrated a superior overall survival rate of 55% in contrast to Black patients, who exhibited a lower rate of 40%, indicating the notably disparate survival outcomes between the two racial groups.

Observational Studies Without Controls

Amber et al. [19] conducted a study involving 290 adult beachgoers who completed a 10-item survey. Each item in the survey featured two different pictures depicting similar skin conditions in individuals of Fitzpatrick skin types I-V. The selection of these images was based on the consensus of five dermatologists and dermatology residents, with an average score exceeding 7.5 for malignant lesions and falling below 2.5 for benign conditions. Malignant conditions depicted included superficial spreading melanoma, acral lentiginous melanoma, basal cell carcinoma, and squamous cell carcinoma, while benign conditions encompassed seborrheic keratosis, solar lentigo, hemangioma, keloid, and verruca vulgaris. Participants were asked to indicate their race and rate their degree of concern regarding whether the pair of images represented a malignant condition on a scale of 1-10. Malignant lesions were scored at 6.99, whereas benign lesions received a rating of 4.42. Notably, participants exhibited a higher degree of concern when inspecting melanoma images (7.25) compared to non-melanoma skin cancers (6.82). Specifically, African American participants assigned a mean score of 6.39 to melanoma images, while non-Hispanic White individuals assigned a mean score of 7.38.

Chapman et al. [20] conducted a survey involving 150 students from three middle schools in South Los Angeles, comprising 96 Latinos and 54 African American youth. The study was initiated with a pre-intervention survey, designed at an adolescent-friendly literacy level, to assess skin cancer/disease awareness and misconceptions. The results from this survey were subsequently analyzed to evaluate dermatologic health literacy. These findings were then condensed into core learning concepts about sun safety and ethnic diversity, which were integrated into an adolescent-appropriate sun safety protection pamphlet. The study employed these finalized pamphlets to deliver a full-day education on skin disease and sun protection to the participants at an early age. Additionally, participants were provided with Cetaphil 30 SPF sunscreen. Subsequently, the study followed up with 148 participants for a three-month post-intervention assessment, aiming to evaluate their knowledge of sun safety, skin cancer, risk, compliance with sun protection products, and their intentions for future use. An interactive presentation was conducted during the post-intervention follow-up, covering various sun safety factors, and Cetaphil 30 SPF sunscreen was distributed to the participants again. Findings revealed that a higher percentage of Latino students (75%) experienced sunburn in the past year compared to African American students (39%). Furthermore, African American students reported using sunscreen "every day" at a rate of 8%, in contrast to Latino students (24%) during the three-month post-intervention follow-up. The study also highlighted that more Latino students expressed their intention to wear sunscreen in the future (97%) compared to African American students (89%).

Wyant et al. [21] conducted an electronic survey involving 859 members and alumni of five Florida marching bands, with 74.6% non-Hispanic White and 3.5% non-Hispanic Black participants. The survey aimed to assess various factors, including demographic characteristics, Fitzpatrick skin type, sun protection behaviors, history of sunburns, and skin cancers. The study also compared sun protection practices between current band members and alumni. The findings indicated that more current band members wore sunscreen (27.1%) during sunny practice days than alumni (16.1%). Alumni were also less likely to wear hats or sunglasses compared to current band members. While the majority of both groups reported using 30 SPF sunscreen, it was noted that they rarely reapplied sunscreen every two hours. A significant proportion of participants (54.3%) had family members with a history of skin cancer, and non-Hispanic White participants were more likely to report having experienced a painful sunburn lasting a day or more compared to non-Hispanic Black participants. Skin cancer cases among the participants, including basal cell carcinoma, squamous cell carcinoma, and melanoma, were predominantly found in non-Hispanic White individuals.

Future research should aim to address these limitations by incorporating specific control groups, such as null or placebo groups, to enable more accurate comparisons and evaluations of educational interventions. Replicating these studies with larger sample sizes and improved follow-up rates would enhance the generalizability of the findings and provide more robust evidence for melanoma interventions. Moreover, the stratification of data by age, stage at diagnosis, and other pertinent variables, as suggested by the studies, would offer a more comprehensive understanding of how these factors influence melanoma outcomes. Future studies should also consider the impact of socioeconomic status, education, and other confounding factors. Diversifying interventions across regions with varying populations and climatic conditions would provide insights into how geographical factors influence sun-protective behaviors and melanoma risk. Finally, conducting long-term observations over extended periods would help assess whether increased skin self-examinations lead to reduced melanoma mortality rates. Long-term studies could provide more robust evidence for the efficacy of interventions. Addressing these limitations and exploring these areas in future research will contribute to a more comprehensive understanding of melanoma prevention, early detection, and treatment strategies across diverse racial and ethnic groups. It is important to acknowledge that the studies mentioned have certain limitations, including small sample sizes, the absence of control groups, recall bias, and selection bias.

## Conclusions

These studies have emphasized the positive impact of targeted educational interventions. This included an improvement in knowledge, self-efficacy, and sun-protective behaviors related to melanoma, with their effectiveness extending to various racial groups, including non-Hispanic Black individuals. Additionally, they analyzed the tendency of African Americans to underestimate the risk of melanoma due to its lower prevalence within their demographic. Therefore, incorporating early melanoma photographs tailored to African Americans can help enhance identification, self-examination practices, and early detection. Furthermore, they illuminated the existing disparities in melanoma incidence and survival rates across different racial groups. Their research emphasized the significance of addressing these racial disparities in terms of melanoma awareness, diagnosis, and treatment. It also highlighted the barriers to sun protection behaviors and the role of health literacy in ethnic communities and younger individuals. These barriers include cost, limited awareness, and misconceptions about sun protection, which significantly contribute to reduced compliance with sun-protective behaviors. Consequently, improving health literacy and addressing these barriers become pivotal in minimizing the risk of melanoma in Black people. 

## Appendices

**Table 2:** Study designs of the seven studies included in this review

Study Design	Number of Studies
Clinical Trials	1
Non-randomized Trial with Controls	1
Observational Studies with Controls	2
Observational Studies Without Controls	3

**Table 3:** Level of evidence in the study The level assigned to the studies for the type of evidence included [21].

Level	Type of study
0	Preclinical studies (including experimental studies and animal models)
1	Randomized controlled trials
2	Non-randomized controlled trial (a prospective study with predetermined eligibility criteria and outcome measures)
3	Observational studies with controls (includes retrospective, case-control studies, and cohort studies
4	Observational studies without controls (includes cohort studies without controls, case series without controls, and case studies without controls)
